# Membrane-Permeable Calpain Inhibitors Promote Rat Oral Mucosal Epithelial Cell Proliferation by Inhibiting IL-1α Signaling

**DOI:** 10.1371/journal.pone.0134240

**Published:** 2015-07-31

**Authors:** Makoto Kondo, Masayuki Yamato, Ryo Takagi, Hideo Namiki, Teruo Okano

**Affiliations:** 1 Institute of Advanced Biomedical Engineering and Science, Tokyo Women’s Medical University, TWIns, 8–1 Kawada-cho, Shinjuku-ku, Tokyo, 162–8666, Japan; 2 Graduate School of Advanced Science and Engineering, Waseda University, 2–2 Wakamatsu-cho, Shinjuku-ku, Tokyo, 162–8480, Japan; Georgetown University, UNITED STATES

## Abstract

To standardise regenerative medicine using cultured cells, the use of serum-free, chemically defined media will be necessary. We have reported that IL-1α inhibits the growth of epithelial cells in culture and that recombinant IL-1 receptor antagonist (IL-1RA) significantly promotes epithelial cell growth in no feeder layer condition. In this study, we examined inhibitors of calpain, a cysteine proteinase that plays crucial roles in various cellular functions, including IL-1α maturation and secretion. The culturing of epithelial cells in serum-free media supplemented with a membrane-permeable calpain inhibitor significantly promoted growth while suppressing IL-1α maturation and secretion. By contrast, non-membrane-permeable calpain inhibitor treatment did not have these effects. Interestingly, immunoblotting analysis revealed that immature, untruncated, IL-1α expression was also downregulated by cell-permeable calpain inhibitor treatment, and the difference in IL-1α gene expression increased from day 2 to day 6. Although IL-1RA has been reported to promote epithelial cell growth, we detected no synergistic promotion of epithelial cell growth using a calpain inhibitor and IL-1RA. These findings indicate that calpain inhibitors promote epithelial cell proliferation by inhibiting IL-1α maturation at an early phase of epithelial cell culture and by suppressing the positive feedback-mediated amplification of IL-1α signalling.

## Introduction

Following the establishment of the human epidermal keratinocyte culture method using foetal bovine serum (FBS) and a 3T3 feeder layer [1, 2], fabricated epidermal cell sheets have been used as epidermal grafts to treat skin defects, such as severe burns [3], ulcers [4] and giant congenital nevi [5]. This culture method has also been applied to oral mucosal epithelial cells [6] and used clinically to treat skin [7, 8] and oral defects [7, 9, 10]. We have also treated corneal defects with transplantable cell sheets fabricated from autologous oral mucosal epithelial cells cultured using FBS and a 3T3 feeder layer [11].

However, the possibility of pathogen transmission or infection from these xenogeneic materials cannot be eliminated. We have reported that cell culture inserts with micropores (0.4 μm) on the bottom promote the proliferation and stratification of canine oral mucosal epithelial cells, even in the absence of both a feeder layer and serum [12]. However, the proliferation of primary human oral mucosal epithelial cells was found to be poor and unstable under these culture conditions. Therefore, transplantable epithelial cell sheets that were fabricated from the autologous oral mucosal epithelium in media supplemented with autologous serum in the absence of a feeder layer have subsequently been used to treat oesophageal ulcers after endoscopic cancer resection [13].

If serum-free culture conditions could be used to fabricate transplantable human epithelial cell sheets, such an approach could yield benefits to patients by avoiding the stress of blood collection and the variance in serum quality between patients.

To develop serum-free culture conditions to fabricate transplantable epithelial cell sheets, factors that promote proliferation should be included as a serum alternative to support the stable culture of epithelial cells. Based on screens for cytokines with epithelial cell proliferation activity, we reported that IL-1α inhibits the growth of epithelial cells, whereas IL-1 receptor antagonist (IL-1RA) promotes growth [14]. This finding suggests that the regulation of endogenous IL-1α signalling might play an important role in epithelial cell proliferation and stem cell maintenance.

Calpain, a Ca^2+^-dependent neutral cysteine proteinase, is known to regulate various cellular functions *in vitro* and *in vivo* [15, 16]. The immature 33-kDa pro-form of IL-1α is converted to the mature 17-kDa form via cytoplasmic calpain activity in various cell types [17–19]; importantly, only the mature 17-kDa form of IL-1α is secreted [18].

Therefore, we hypothesised that calpain might play an important role in epithelial cell growth. Herein, we assessed the cell proliferative effects of various calpain inhibitors applied as a supplement to serum-free culture medium.

## Materials and Methods

### Isolation of oral mucosal epithelial cells

Experimental animals were treated in accordance with experimental procedures approved by the Committee for Animal Research of Tokyo Women’s Medical University in Tokyo, Japan. Totally 22 rats were used in this study. After the humane euthanasia with CO_2_, Lewis rats (8 weeks old, male, from Charles River, Wilmington, MA), the oral mucosal tissues were surgically excised from buccal mucosa, disinfected with povidone-iodine (Meiji Seika Pharma, Tokyo, Japan), and washed with Dulbecco’s Modified Eagle Medium (DMEM; Sigma-Aldrich, St Louis, MO) containing 100 IU/mL penicillin and 100 μg/mL streptomycin (Life Technologies, Carlsbad, CA, USA). The oral mucosal tissues were digested with 1000 PU of dispase (Godo Shusei, Tokyo, Japan) at 4°C overnight, and then, the epithelial tissue was “peeled off” using forceps. The epithelium was then torn using forceps and was dissociated using 1.25% trypsin-0.5% ethylenediaminetetraacetic acid (EDTA) in Dulbecco’s phosphate buffered saline (Sigma-Aldrich) at 37°C for 15 min to obtain epithelial cell suspensions. Disaggregated cells were filtered through 40-μm cell strainers (BD Biosciences, Franklin Lakes, NJ). Enzymatic treatment was stopped by adding a trypsin inhibitor (DS Pharma Biomedical, Osaka, Japan), and the cells were then cultured.

### Cell culture

Keratinocyte culture medium (KCM) was composed of a basal mixture of 3 parts DMEM to 1 part nutrient mixture F-12 Ham (Sigma-Aldrich) supplemented with 2 nM triiodothyronine (Wako Pure Chemicals, Osaka, Japan), 0.5% insulin-transferrin-selenium reagent (Life Technologies), 5 μg/ml transferrin (Life Technologies), 10 ng/mL epidermal growth factor (Protein Express, Chiba, Japan), 0.4 μg/mL hydrocortisone (Kowa Pharmaceutical, Tokyo, Japan), 1 nM cholera toxin (List Biological Labs, Campbell, CA), 0.5 μg/mL amphotericin B (Fungizone; Bristol-Myers Squibb, Park Avenue, NY), and 40 μg/mL gentamicin (Gentacin; Schering-Plough, Kenilworth, NJ).

### Epithelial cell culture in the presence of calpain inhibitors

Isolated primary rat oral mucosal epithelial cells were cultured on cell culture inserts (23-mm in diameter; BD Biosciences) at an initial density of 4.0 × 10^4^ cells/cm^2^ in KCM without a 3T3 feeder layer at 37°C in a humidified atmosphere containing 5% CO_2_. Cell morphology and proliferation were monitored using a phase-contrast microscope (ECLIPSE TE2000-U, Nikon, Tokyo, Japan). We evaluated the effects of the following calpain inhibitors: calpeptin (50 nM; MW: 362.5, ENZO Life Sciences, Farmingdale, NY) calpain inhibitor III (4 nM; MW: 382.5, Calbiochem, San Diego, CA), calpastatin (50 nM; MW: 14000, Takara Bio, Shiga, Japan), B27-WT (1 μM; MW: 3136.6, AnaSpec, Fremont, CA), and cystatin C (15 nM; MW: 15 kDa, R&D Systems, Minneapolis, MN). For specific experiment, 1 μg/mL IL-1RA or 10 ng/mL IL-1α was exogenously added. The utilised concentrations of these inhibitors and cytokines were determined based on preliminary experiments and previous study [14].

### Cell number quantification

**Fig 1:**
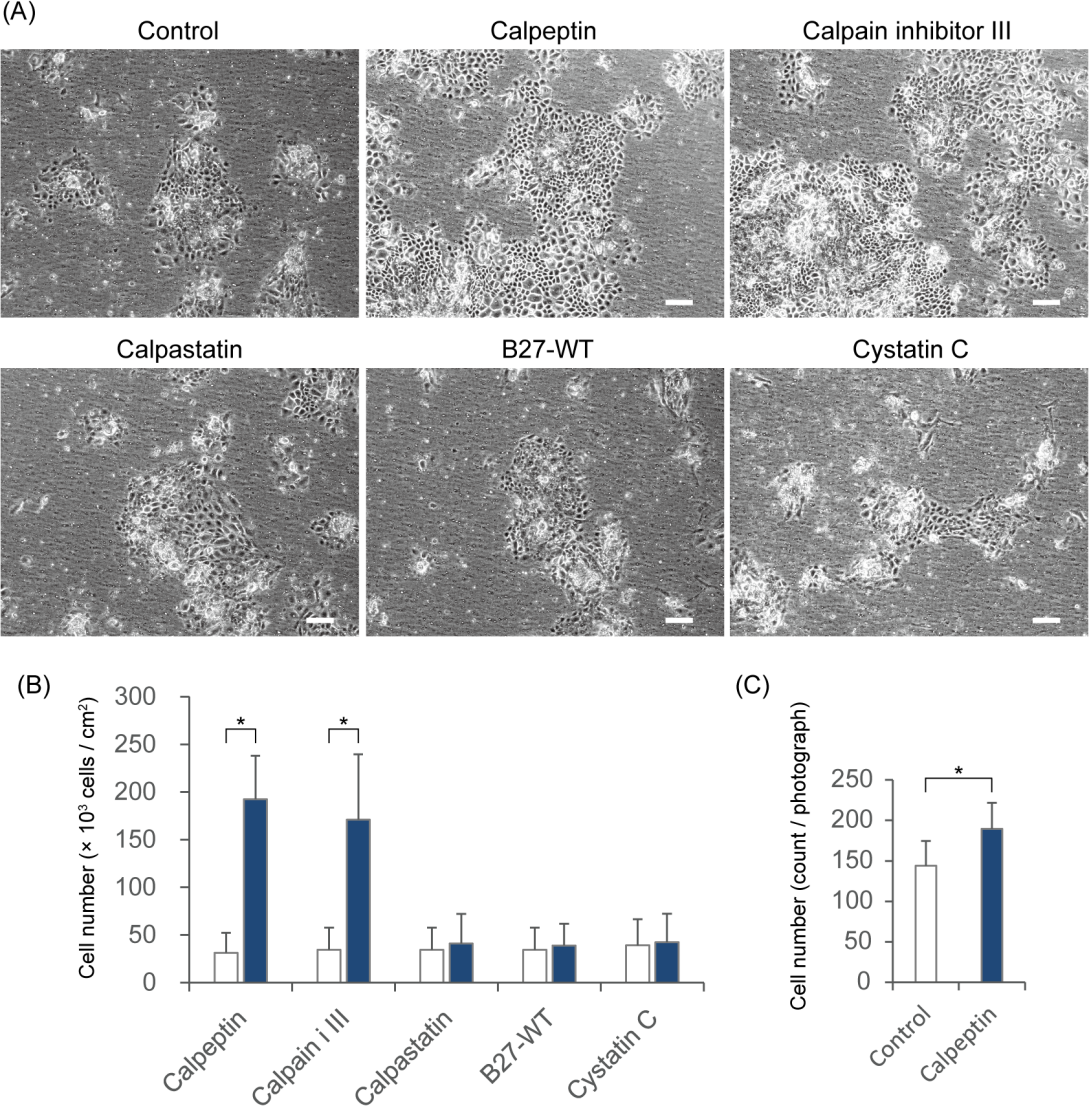
The proliferation and attachment of oral mucosal epithelial cells in the presence of calpain inhibitors. (A) Phase contrast microscopic images of cultured rat oral mucosal epithelial cells. The bars indicate 200 μm. (B) The cells were collected and counted at 8–11 days after seeding (calpeptin, *n* = 4; calpain inhibitor III, *n* = 3; calpastatin, *n* = 3; B27-WT, *n* = 3; and cystatin C, *n* = 2). White blocks, controls; blue blocks, inhibitors. * *P* < 0.05 using Student's *t*-test. (C) The number of initially attached epithelial cells was determined 48 h after seeding (*n* = 4). The error bars indicate the SD. * *P* < 0.05 using Student's *t*-test.

Initial cell attachment at 48 h (Fig 1C) was quantified by counting the cells in randomly taken 15 images (*n* = 4). The images were taken after several time washing with warmed KCM. For quantification of cultured cells, the cells were harvested by using 1.25% trypsin-0.5% EDTA when the most rapid growth condition reached 90% confluence (8–11 days).

#### Quantification of IL-1α concentrations in conditioned medium

Supernatants from the cells subjected to different culture conditions were individually collected and examined via enzyme-linked immunosorbent assay (ELISA). All supernatants that were evaluated for IL-1α secretion were from cultures that were incubated in KCM in the presence or absence of a calpain inhibitor. The media were changed once every few days, and the culture supernatants were collected at 8 or 9 days after seeding. The amounts of secreted IL-1α were quantified using a specific Quantikine ELISA kit (R&D Systems) according to the manufacturer’s protocol.

### Immunoblotting

The cells were treated with 10% w/v trichloroacetic acid (Sigma-Aldrich) in saline on ice for 30 min at 9 days after seeding. Whole cell lysates were treated with NuPAGE LDS sample buffer (Life Technologies) and then subjected to electrophoresis using a gradient gel (Nu-PAGE 4–12% Bis-Tris Gel, Life Technologies). The resolved proteins in the gel were electrophoretically transferred to nitrocellulose membranes (iBlot Gel Transfer Stack, Life Technologies), which were then incubated at room temperature for 1 h in blocking buffer composed of 5% skim milk (Cell Signaling Technology, Danvers, MA) in 20 mM Tris-buffered saline (pH 7.5) containing 0.1% Tween-20 (TBS-T). After blocking, the membranes were treated with one of the following primary antibodies: goat polyclonal anti-IL-1α (1:10,000 dilution in 5% BSA containing TBS-T; PeproTech), rabbit monoclonal anti-calpain I (1:2000 dilution in 5% BSA containing TBS-T; Cell Signaling Technology), rabbit monoclonal anti-calpain II (1:2000 dilution in 5% BSA containing TBS-T; Cell Signaling Technology), or rabbit monoclonal anti-β-actin (1:2000 dilution in 5% BSA containing TBS-T; 13E5, Cell Signaling Technology) at 4°C. After incubation overnight, the membranes were washed with TBS-T and then incubated with an HRP-conjugated anti-mouse or anti-rabbit IgG secondary antibody (1:20,000 dilution in blocking buffer; Cell Signaling) at room temperature for 1 h. Immunoreactive bands corresponding to the IL-1α, calpain, and β-actin proteins were detected using ECL Prime Western Blotting Detection Reagent (GE Healthcare).

### Gene expression analysis

Rat oral mucosal epithelial cells were seeded at a density of 4.0 × 10^4^ cells/cm^2^ and were cultured for 2, 4, 6, 8, or 10 days. RNA was extracted from cultured cells using an RNeasy Plus Mini Kit (Qiagen, Hilden, Germany). Single-stranded cDNA was synthesised via a reverse transcription reaction using a PrimeScript RT reagent Kit (Takara Bio) in an iCycler Thermal Cycler (Bio-Rad Laboratories, Hercules, CA). The gene expression levels were quantified using a TaqMan Fast Universal PCR Master Mix and a StepOnePlus Real-Time PCR System (Life Technologies). Primer pairs and TaqMan MGB probes were designed for B2M, IL1α, IL1RA, TNFα, p63, Bmi1, IL1RI, IL1RII, calpain I, and calpain II for TaqMan Gene Expression Assays (Life Technologies). The mRNA expression levels were normalised to that of β2-microglobulin. The data obtained from measurements of three independent cultures were statistically analysed using Student's *t*-test.

## Results

### Calpain inhibitors promote epithelial cell growth

Calpain inhibitors of various molecular weights were added to cultures of primary rat oral mucosal epithelial cells, which were seeded at a density of 4.0 × 10^4^ cells/cm^2^ on cell culture inserts in KCM without FBS or a 3T3 feeder layer. Treatment with a low molecular compound membrane-permeable calpain inhibitor (calpeptin or calpain inhibitor III) strongly promoted cell proliferation (Fig 1A and 1B). However, higher molecular weight peptide or protein (membrane-impermeable) calpain inhibitors (B27-WT, calpastatin, and cystatin C) had no effect on cell proliferation (Fig 1A and 1B). To determine whether calpain is involved in the early process of cell attachment, cells were seeded at a density of 4.0 × 10^4^ cells/cm^2^, and the number of attached cells was counted prior to colony formation. Cell attachment was slightly promoted only in the presence of calpeptin (Fig 1C). Therefore, the calpain inhibitor-mediated promotion of epithelial cell growth is probably not mainly due to an early promotion of cell attachment.

### Calpain inhibitors reduce IL-1α secretion

**Fig 2:**
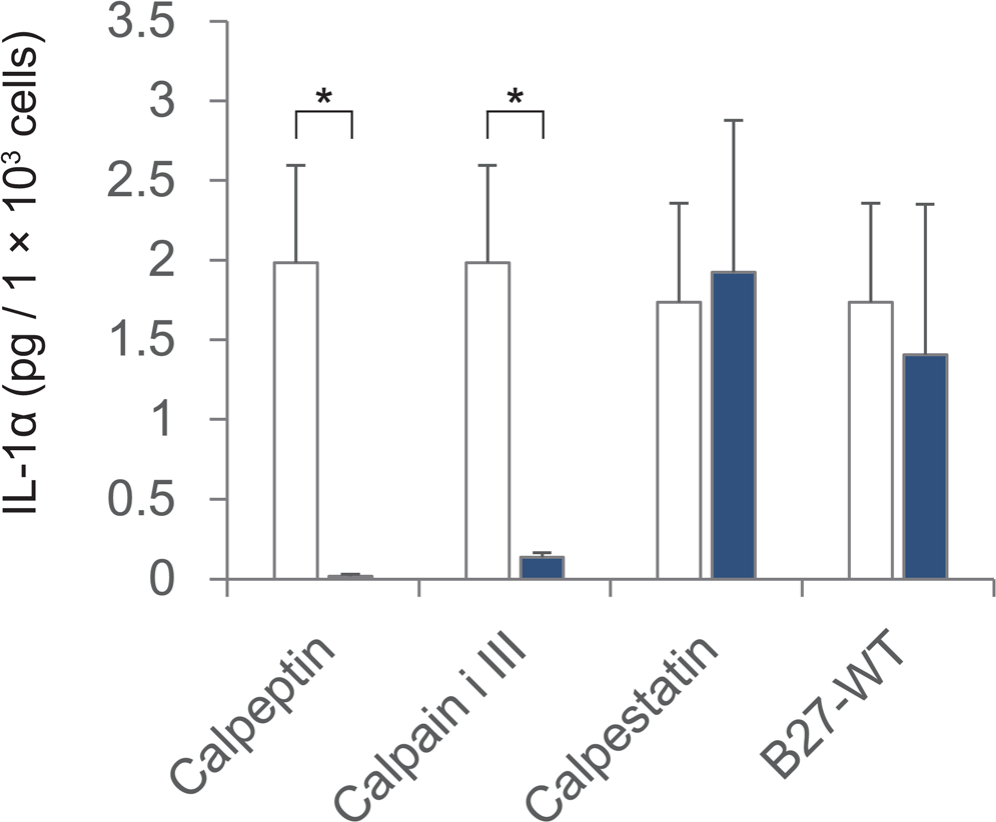
IL-1α levels in the supernatant of epithelial cell cultures. IL-1α was measured in supernatants of rat oral mucosal epithelial cell cultures via ELISA. The amount of supernatant assessed on day 8 or 9 was adjusted based on the number of cells for each condition. Calpeptin, *n* = 3; calpain inhibitor III, *n* = 3; calpastatin, *n* = 2; and B27-WT, *n* = 2. White blocks, controls; blue blocks, inhibitors. The error bars indicate the SD. * *P* < 0.05 using Student's *t*-test.

Cultured oral mucosal epithelial cells constitutively express IL-1α [20, 21]. Moreover, cytoplasmic calpain is known to cleave pro-IL-1α to mature IL-1α, which can be secreted [17, 18]. Furthermore, we recently reported that exogenous IL-1α treatment reduces epithelial cell growth [14]. Therefore, we firstly hypothesised that a cell culture environment containing membrane-permeable calpain inhibitors might promote epithelial cell growth by inhibiting the maturation and secretion of endogenous IL-1α. The concentration of IL-1α in the conditioned medium was measured via ELISA of the cell culture supernatants. The concentration of IL-1α in the conditioned medium was lower in the presence of membrane-permeable calpain inhibitors than in the other culture conditions. In cultures treated with a low-molecular-weight calpain inhibitor, such as calpeptin or calpain inhibitor III, the release of IL-1α was potently inhibited (Fig 2). By contrast, IL-1α release was not inhibited by the high-molecular-weight membrane-impermeable calpain inhibitors (Fig 2). These results indicate that the inhibition of cytoplasmic calpain proteolytic activity suppressed endogenous IL-1α maturation, reduced the secretion of mature IL-1α, and suppressed the IL-1α-mediated inhibition of epithelial cell growth in culture.

### Detection of cellular IL-1α expression via immunoblotting

**Fig 3:**
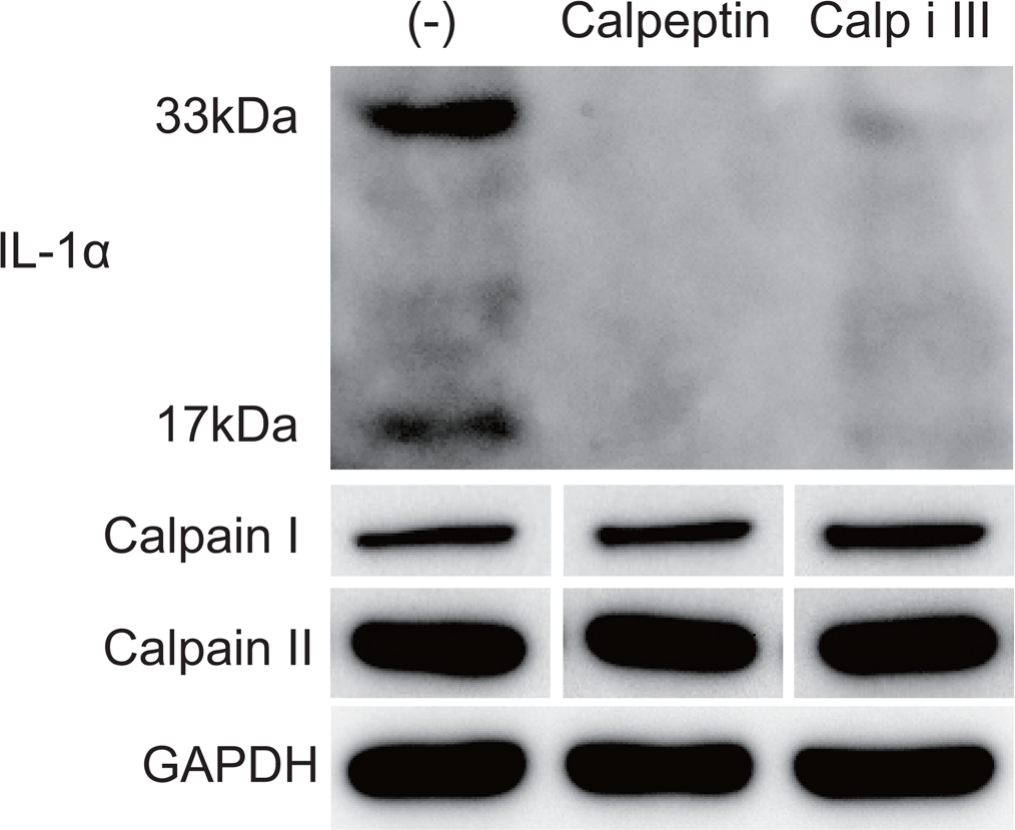
Expression levels of cellular IL-1α and calpains. The expression levels of the 33-kDa pro-IL-1α, the 17-kDa mature IL-1α, calpain I, and calpain II were detected in cultured rat oral mucosal epithelial cells via immunoblotting. GAPDH expression was used as an internal control. The cultured epithelial cells were harvested on day 8.

Immunoblotting of cell lysates using an anti-IL-1α antibody revealed that a small amount of mature IL-1α (17 kDa) accumulated in the cytoplasm in the presence of membrane-permeable calpain inhibitors (Fig 3 and S1–S4 Figs). Interestingly, the accumulation of the untruncated form of IL-1α (33 kDa) was also suppressed. By contrast, the expression levels of calpain I and II were not affected by calpain inhibitor treatment.

### Gene expression kinetics

**Fig 4:**
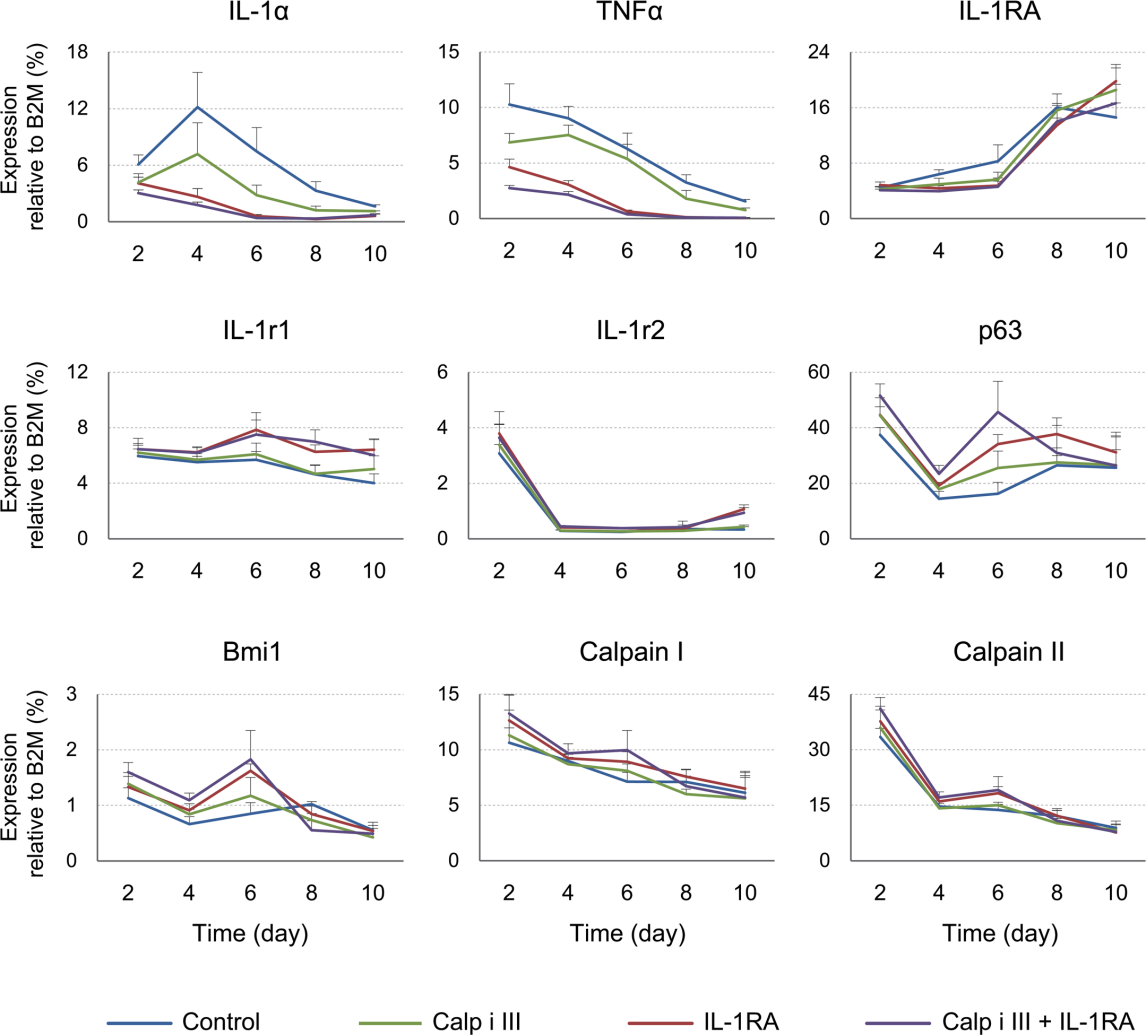
Time-course of gene expression in cultured oral mucosal epithelial cells. The gene expression levels of IL-1α, TNF-α, IL-1RA, calpain I, calpain II, IL-1 receptor 1 (IL-1r1), IL-1 receptor 2 (IL1r2), p63, and Bmi1 were quantified via qRT-PCR. Primary cultures of rat oral mucosal epithelial cells were harvested on day 2, 4, 6, 8, or 10 (*n* = 4). β-2-microglobulin gene expression was used as an internal control. The error bars indicate the SD.

To investigate the periodical effects of the calpain inhibitors on IL-1α gene expression, time-course qPCR was performed on cultured oral mucosal epithelial cells. Also, recombinant IL-1RA was exogenously added as a binding blocker of IL-1α to the IL-1 receptor, inhibiting IL-1 signalling [22, 23]. The time-course of IL-1α gene expression is shown in Fig 4. Under control conditions in the absence of a membrane-permeable calpain inhibitor, the gene expression of IL-1α increased from day 2 to 4 and then decreased. TNF-α expression declined throughout the culture period, and IL-1RA expression increased throughout the culture period under all culture conditions. Additionally, calpain inhibitor III and IL-1RA significantly attenuated the expression of the IL-1α and TNF-α genes. The inhibitory effect of IL-1RA was stronger than that of calpain inhibitor III. Treatment with both calpain inhibitor III and IL-1RA resulted in a similar downregulatory effect to that of IL-1RA treatment alone. The expression of the IL-1 receptor II, calpain I, and calpain II genes was unchanged. The expression of IL-1 receptor I after treatment with IL-1RA was slightly but significantly increased. The expression of p63 and Bmi1, epithelial stem/progenitor markers, was modestly but significantly increased in cultures treated with calpain inhibitor III, IL-1RA, or both at days 2, 4, and 6.

### IL-1RA and calpain inhibitors do not exert synergistic effects on epithelial cell growth

**Fig 5:**
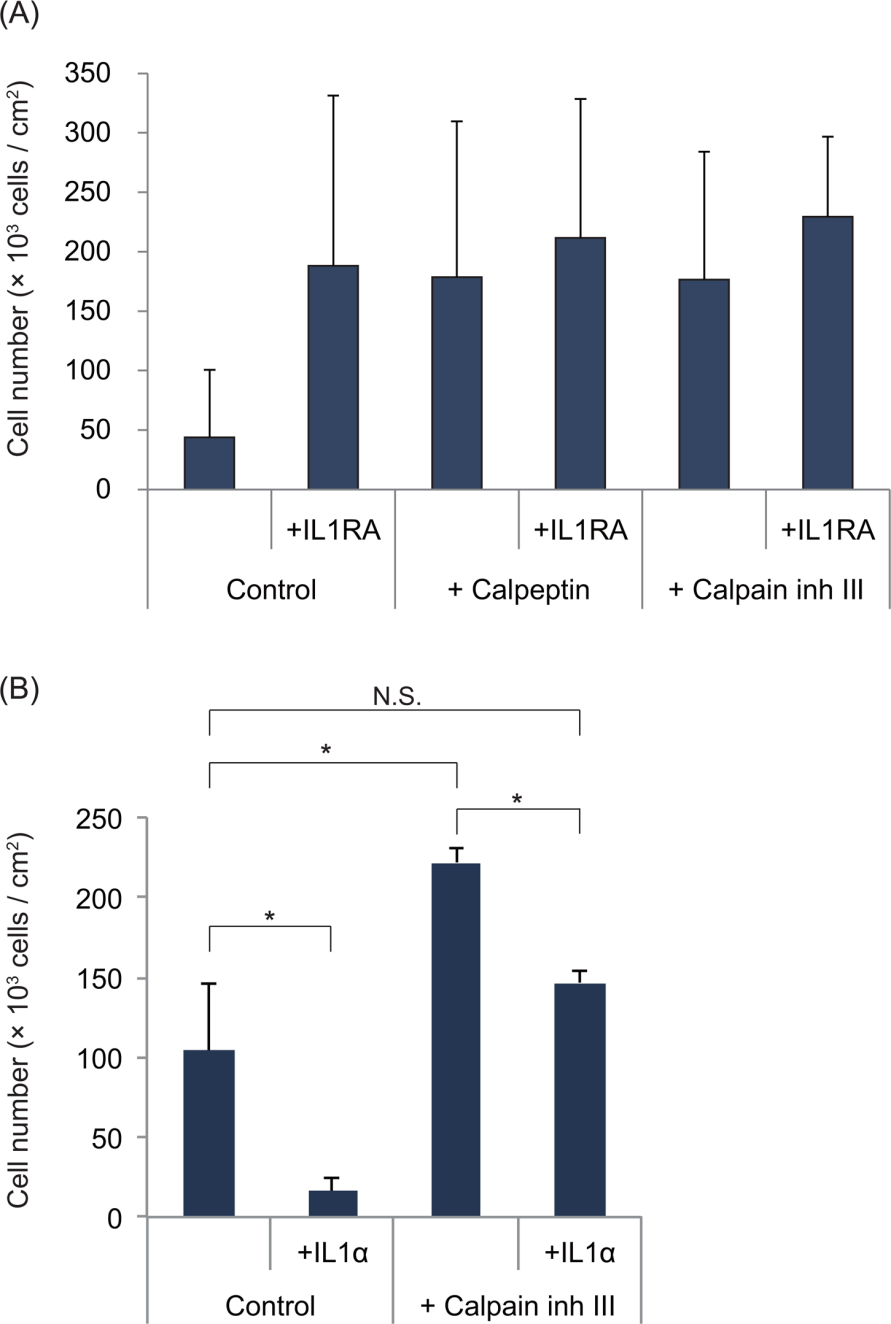
Treatment of cell cultures with a calpain inhibitor (A) and/or IL-1RA (B) and/or IL-1α. The reagents were applied upon seeding the cells, and the cells were collected and counted on (A) day 7 or 11 (*n* = 3) (B) day10 (n = 3). The error bars indicate the SD. (A) No significant differences were detected between any of the reagent-treated cultures. (B) * *P* < 0.05 using Student's *t*-test. N.S. indicates non-significant difference.

Based on the expression of the immature and mature protein and mRNA encoding IL-1α, we hypothesised that the cell growth promotion effect of calpain inhibitors is related to the regulation of IL-1α expression. Therefore, we added IL-1RA to cultures treated with a calpain inhibitor to confirm the proliferation-enhancing effect of the calpain inhibitors (Fig 5A). Calpain inhibitor and/or IL-1RA treatment significantly promoted cell proliferation compared to the control conditions. However, no significant difference was detected between treatment with either a calpain inhibitor or IL-1RA alone and treatment with both compounds.

To clarify calpain inhibitor’s effect on IL-1α signaling, exogenous IL-1α was added to calpain inhibitor-containing condition (Fig 5B). Exogenous IL-1α dramatically inhibited epithelial cell growth as we have shown in previous study [14]. In calpain-inhibiting condition, exogenous IL-1α cancelled the growth promoting effect of calpain inhibitor, indicating that calpain inhibition acts by reducing IL-1α signaling.

## Discussion

The roles of calpain in cellular signalling have been widely investigated. However, the effect of calpain inhibition on epithelial cell proliferation has not been reported. To make cell therapy using epithelial cells a viable option, a chemically defined serum-free culture system should be established, even though serum depletion sharply reduces cell growth in high Ca^2+^-containing media.

In this study, we found that inhibition of cytoplasmic calpain-mediated proteolytic activity using a membrane-permeable inhibitor significantly promoted epithelial cell proliferation, even under serum-free culture conditions (Fig 1). Calpeptin inhibits not only calpain I and II but also cathepsin K and L. Calpain inhibitor III, a specific inhibitor of calpain I and II, also exerted growth-promoting effects (Fig 1). However, a membrane-permeable cathepsin-specific inhibitor (Cathepsin K Inhibitor I, Merck, Darmstadt, Germany) had no growth-promoting effect (data not shown). Therefore, cytoplasmic calpain is most likely responsible for the negative regulation of epithelial cell growth via IL-1α maturation. Calpain I, referred to as μ-calpain, displays proteolytic enzyme activity when Ca^2+^ concentrations are in the micro molar range or higher. Calpain II, also known as m-calpain, is active when Ca^2+^ concentrations are in the mill molar range or higher. KCM contains 1.095 mM Ca^2+^. Thus, both calpains can be active in the extracellular environment, but cell-impermeable calpain inhibitors were unable to increase cell proliferation (Fig 1). Therefore, cytoplasmic calpain is most likely responsible for the promotion of epithelial cell proliferation. The cytoplasmic free [Ca^2+^] and molecular-bound [Ca^2+^] are approximately 100 nM and 10 μM, respectively, under physiological conditions [24]. However, these concentrations can be promoted by certain components in the medium. It is difficult to determine whether calpain I or II more effectively regulates the growth of cultured epithelial cells.

Calpain converts immature IL-1α into mature IL-1α, and exogenously added IL-1α inhibits epithelial cell growth. Therefore, we first hypothesised that this effect of calpain occurs through its effects on IL-1α maturation and secretion. However, surprisingly, the expression of both the mature (17 kDa) and immature (33-kDa) forms of IL-1α was inhibited by calpain inhibitor treatment for 8 days (Fig 3). This result appeared that calpain could promote the expression of immature IL-1α. Moreover, gene expression difference of IL-1α between the calpain inhibitor-treated condition and control was increased on days 4 and 6 compared to day 2 (Fig 4). Furthermore, intracellular IL-1α protein was initially not detectable because of its low abundance under all conditions. This result is possibly due to the inhibition of the unique “positive feedback” regulation of IL-1α signaling [25], as we inhibited calpain upon seeding the cells. Calpain inhibitors inhibit the initial step in IL-1α maturation, thus suppressing the accumulation of IL-1α. By contrast, more IL-1α was truncated and secreted of the cultures under control condition, which invokes further positive feedback of IL-1α.

TNF-α strongly inhibits the growth of human neonatal foreskin keratinocytes [26], and we also confirmed this inhibitory effect on rat oral mucosal epithelial cells (data not shown). In this study, TNF-α expression was significantly downregulated by calpain inhibitor treatment (Fig 4). Exogenous IL-1α slightly induces the expression of the TNF-α gene in human epidermal keratinocytes [14], whereas TNF-α induces IL-1α expression in human epidermal keratinocytes [27]. This finding indicates that IL-1α and TNF-α might amplify the expression of one another, thus strongly inhibiting epithelial cell proliferation *in vitro*. The mechanisms by which IL-1α and TNF-α inhibit cell growth have yet to be elucidated, but both cytokines have been reported to activate NF-κB, which arrests the growth of normal human skin epithelial cells [28]. Presumably, calpain inhibition might promote cell growth by suppressing NF-κB signalling via the inhibition of the expression of these cytokines. IL-1RA blocks the IL-1 receptor. IL-1RA expression increased over time under all culture conditions (Fig 4), but its expression occurred too late to suppress the positive feedback of IL-1α expression under the control conditions. The expression of the stem/progenitor markers p63 and Bmi1 was slightly but significantly enhanced at early time points (days 2–6; when the cells were not confluent) due to treatment with a calpain inhibitor and/or IL-1RA (Fig 4). These genes might be involved in the strong cell proliferative effects of these inhibitors at early growth stages. Moreover, p63 and Bmi1 expression were not abnormally increased (Fig 4), and the cells that reached confluence displayed contact inhibition (data not shown). Therefore, the cultured cells probably did not acquire malignant properties and may be useful for therapeutic use.

**Fig 6:**
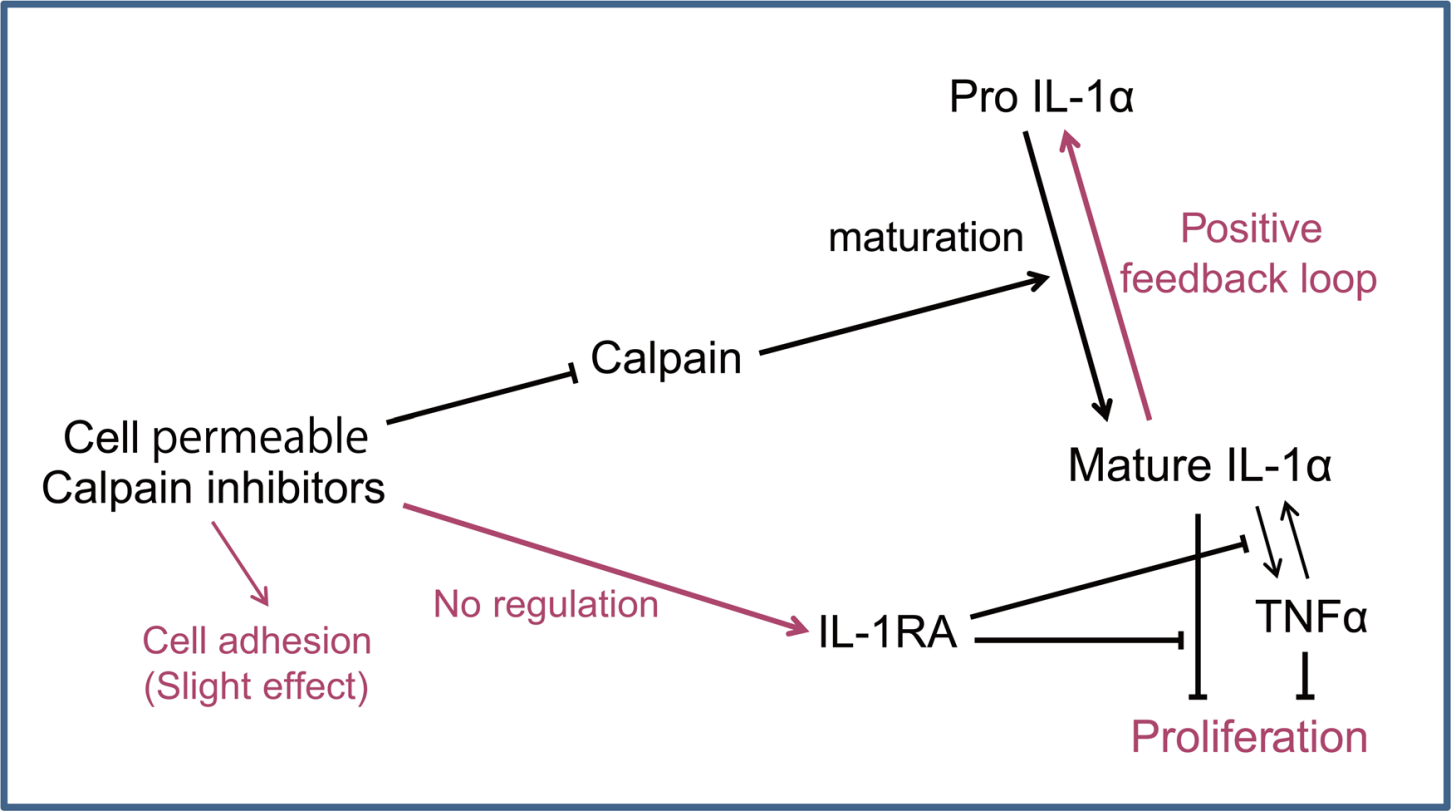
Scheme of calpain and IL-1α signalling in regulating epithelial proliferation by cell permeable calpain inhibitors.

Furthermore, the combined application of IL-1RA and a calpain inhibitor did not significantly promote cell growth compared to application of a calpain inhibitor alone (Fig 5A) and exogenous IL-1α cancelled the calpain inhibitor’s cell growth promotional effect (Fig 5B). These results indicate that calpain inhibition affects cell proliferation by regulating IL-1α. Presumably, there is crosstalk between epithelial cell proliferation pathways and calpain activity, as shown in Fig 6.

The cellular responses to medium components vary between cells from experimental animals and humans [29]. In addition, primary cultures of keratinocytes and commercially available keratinocytes display distinct responses to medium components, such as Ca^2+^ and serum [30], owing to differences in the isolation procedure that affect cell proliferation and differentiation properties. Therefore, oral mucosal epithelial cells from human volunteers and patients should be examined, even though their availability is limited. Further elucidation of the roles of the calpain super-family and of potential calpain inhibitors will be important for evaluating various approaches and will be useful for optimising the epithelial cell culture process for regenerative medicine.

## Supporting Information

S1 Fig Raw data of GAPDH western blotting. (PDF)

S2 Fig Raw data of IL-1α western blotting. (PDF)

S3 Fig Raw data of calpain I western blotting. (PDF)

S4 Fig Raw data of calpain II western blotting. (PDF)
