# Supplementary figures and images for: Membrane-Permeable Calpain Inhibitors Promote Rat Oral Mucosal Epithelial Cell Proliferation by Inhibiting IL-1α Signaling
Source: PLoS One. 2015 Jul 31;10(7):e0134240. doi: 10.1371/journal.pone.0134240 (PMC4521813; doi:10.1371/journal.pone.0134240)

Control Calpeptin Calpain inh III

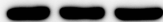

Supplement: S1 Fig — (PDF) [file pone.0134240.s001.pdf]

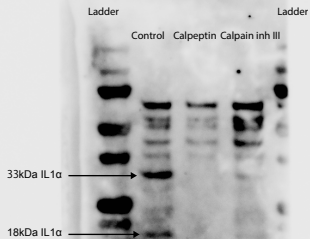

Supplement: S2 Fig — (PDF) [file pone.0134240.s002.pdf]

Non-relevant samples

Control

Calpeptin

Calpain inh III

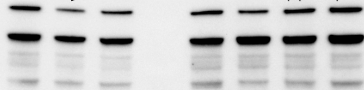

Supplement: S3 Fig — (PDF) [file pone.0134240.s003.pdf]

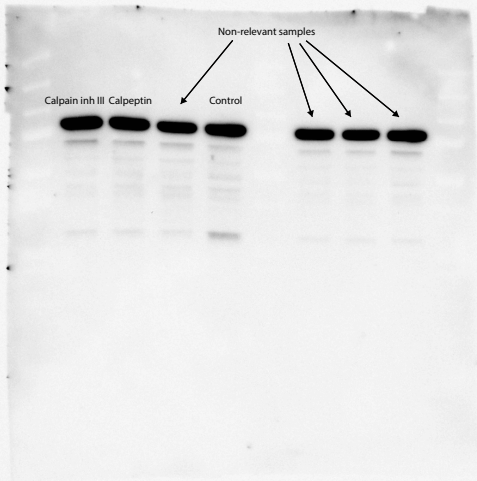

Supplement: S4 Fig — (PDF) [file pone.0134240.s004.pdf]
